# Connecting rules from paired miRNA and mRNA expression data sets of HCV patients to detect both inverse and positive regulatory relationships

**DOI:** 10.1186/1471-2164-16-S2-S11

**Published:** 2015-01-21

**Authors:** Renhua Song, Qian Liu, Tao Liu, Jinyan Li

**Affiliations:** 1 Advanced Analytics Institute, University of Technology, Sydney, Broadway, New South Wales 2007, Australia; 2 Children's Cancer Institute Australia for Medical Research, Randwick, New South Wales 2007, Australia

## Abstract

**Background:**

Intensive research based on the inverse expression relationship has been undertaken to discover the miRNA-mRNA regulatory modules involved in the infection of Hepatitis C virus (HCV), the leading cause of chronic liver diseases. However, biological studies in other fields have found that inverse expression relationship is not the only regulatory relationship between miRNAs and their targets, and some miRNAs can positively regulate a mRNA by binding at the 5' UTR of the mRNA.

**Results:**

This work focuses on the detection of both inverse and positive regulatory relationships from a paired miRNA and mRNA expression data set of HCV patients through a 'change-to-change' method which can derive connected discriminatory rules. Our study uncovered many novel miRNA-mRNA regulatory modules. In particular, it was revealed that GFRA2 is positively regulated by miR-557, miR-765 and miR-17-3p that probably bind at different locations of the 5' UTR of this mRNA. The expression relationship between GFRA2 and any of these three miRNAs has not been studied before, although separate research for this gene and these miRNAs have all drawn conclusions linked to hepatocellular carcinoma. This suggests that the binding of mRNA GFRA2 with miR-557, miR-765, or miR-17-3p, or their combinations, is worthy of further investigation by experimentation. We also report another mRNA QKI which has a strong inverse expression relationship with miR-129 and miR-493-3p which may bind at the 3' UTR of QKI with a perfect sequence match. Furthermore, the interaction between hsa-miR-129-5p (previous ID: hsa-miR-129) and QKI is supported with CLIP-Seq data from starBase. Our method can be easily extended for the expression data analysis of other diseases.

**Conclusion:**

Our rule discovery method is useful for integrating binding information and expression profile for identifying HCV miRNA-mRNA regulatory modules and can be applied to the study of the expression profiles of other complex human diseases.

## Introduction

HCV is a positive sense single-stranded RNA Hepacivirus in the family of Flaviviridae [1]. HCV is capable of infecting human liver with the contagious and potentially life-threatening liver disease Hepatitis C. It is estimated that HCV has infected approximately 170 million people worldwide [2], causing a serious public health problem. The molecular basis of this disease is still under intensive investigation.

miRNAs, small (~22 nucleotides), endogenous and highly conserved non-coding RNA molecules [3], play a pivotal role in cell differentiation, proliferation, growth, mobility, and apoptosis, as well as in viral replication and proliferation such as in HCV infections and replication [4]. miRNAs affect the stability and translational efficiency of target mRNAs by binding to their 3' untranslated regions (UTRs) to inhibit expression [5]. A miRNA can have many target mRNAs and a mRNA can be regulated by multiple miRNAs, forming complicated many-to-many regulatory modules between miRNAs and mRNAs.

The identification of miRNA-mRNA regulatory modules has proven to be important for understanding complex cellular systems [5]. It is also useful for understanding the infection process of human diseases [6]. A recent computational method based on probabilistic learning has been specially designed which uses the paired expression profiles and binding information of miRNAs and mRNAs of human cancer samples to discover miRNA-mRNA modules [7]. Bayesian networks have also been adopted by many research groups [8,9] to detect novel miRNA-mRNA modules.

The key idea in all of these studies is the inverse expression relationship between miRNAs and their target mRNAs. An inverse expression relationship means that when the expression level of the miRNA is high (up-regulated), the target mRNA should be down-regulated based on the principle that miRNAs deregulate the expression of targeted mRNAs [10]. However, up-to-date evidence shows that the inverse relationship does not always hold. First, a miRNA can induce gene expression by binding to the gene's promoter or enhancer sequence. For example, miR-373 can induce the expression of E-Cadherin or *CSDC2 *when binding to these genes' promoters [11]. Second, recent investigation also shows that the interaction of miR-10a with RP mRNAs (those mRNAs encoding ribosomal proteins) binding at their 5' UTRs can promote the translational enhancement of these mRNAs instead of repression [12]. Third, some positively regulated modules of miRNAs and mRNAs have been studied by wet-labs. For example, Enerly *et al*. have reported strong positive correlations between miRNA clusters and their target genes of distinct biological processes in primary human breast tumors [13]. Nazarov *et al*. have identified several interactions in the form of negative or positive correlations between miRNAs and mRNAs, and subsequently identified positively correlated miRNA-mRNA interaction networks in the frontal cortex of mice by differential expression analysis and weighted gene co-expression network analysis [14]. Therefore, the identified miRNA-mRNA interactions based purely on the inverse regulatory relationship are only an incomplete part of the modules in a certain biological context.

Recently, Zhang et al. (2011) developed a framework of sparse network-regularized multiple non-negative matrix factorization (SNMNMF) to discover miRNA-gene comodules based on factorized coefficient matrices by integrating diverse data sources [15]. Le et al. (2013) designed a iterative learning framework of protein interaction-based microRNA modules (PIMiM) by combining sequence, expression and interaction data, which can perform better than previous methods (including SNMNMF) [16]. However, both SNMNMF method and PIMiM method require a predefined dimension of the matrix factorization (approximately equal to the number of modules), which may be difficult to determine beforehand. Additionally, the time complexity of these algorithms is quadratic to both the number of miRNA and mRNA multiplied by the total number of modules per iteration. Li et al. (2014) proposed a novel method called Mirsynergy to detect miRNA regulatory modules (MiRMs) by integrating mRNA/miRNA expression profiles, target site information and gene-gene interaction to form MiRMs, which can automatically determine the module number and improve the time complexity [17]. However, these prediction methods often focused on the anti-correlation modules and ignored the positive regulatory relationship in the identified modules.

This work focuses on the detection of both inverse and positive regulatory relationships in the paired miRNA and mRNA expression data of HCV-affected tissue samples. Paired miRNA and mRNA expression profiling provides an excellent platform for capturing those miRNA expression changes between two classes of samples that lead, positively or negatively, to the changes in mRNA expressions between the two classes of samples. We present a novel two-step sequential method to capture such 'changes-to-changes'. Our method derives discriminatory rules from miRNA expression data as the first step, and derives discriminatory rules from mRNA expression data as the second step. These rules are then combined to discover miRNA-mRNA regulatory modules.

**Figure 1:**
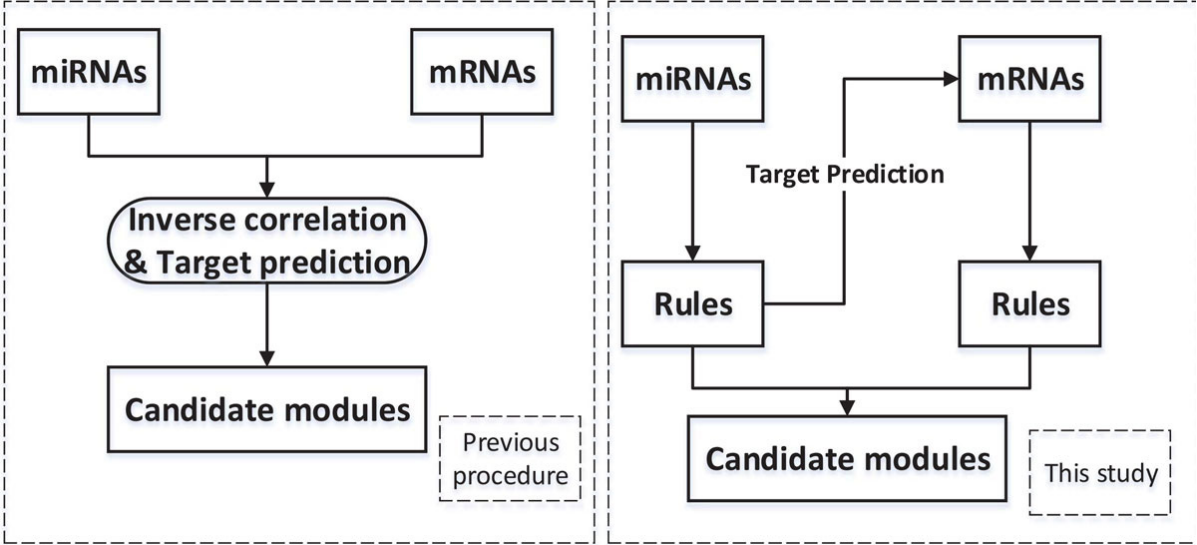
**Our approach in comparison to previous approaches**. We construct miRNA-mRNA regulatory modules using rule-based methods as shown in the right panel where the mRNA data set is narrowed down by the identified miRNA rules which are derived at the first step.

The first step works on the miRNA data of the HCV negative and positive tissue samples to derive differentially expressed miRNAs and discriminatory rules (i.e., the miRNA expression changes between the two classes of samples). For each of these rules, we search for the predicted mRNA targets of every miRNA from the public miRNA target database TargetScan [18]. We then narrow the search findings to a selected mRNA data set by removing the expression data of those mRNAs which do not belong to the predicted target mRNAs from the original mRNA data set. Discriminatory rules are derived from this selected and relevant data set of mRNA expression to concentrate on gene expression patterns that show significant differences between HCV positive and negative tissue samples (i.e., the mRNA expression changes led by those miRNA expression changes detected in the first step). Then, all the miRNAs in a rule and the mRNAs involved in the mRNA rules are combined to form a potential miRNA-mRNA regulatory module which is subsequently analyzed using Pearson's correlation coefficients and biological literature results. Our approach does not use expression similarity networks or gene clusters to connect the two expression data sets, which differs fundamentally from the traditional approaches [6,7,19,20] (see Figure 1 for detailed description).

## Methods

### miRNA and mRNA expression data sets

The HCV data set from Peng *et al*. (2009) is used in this study (downloaded from the NCBI (National Center for Biotechnology Information) GEO (Gene Expression Omnibus) database under the SuperSeries accession number GSE15387). This data set contains 36 tissue samples (24 HCV positive/+ and 12 HCV negative/-) described by the expression levels of 470 human miRNAs and 22575 mRNAs. The miRNA and mRNA data sets were both pre-processed using the Agilent Feature Extraction v9.5.3 under the default miRNA or mRNA parameters. Each miRNA value is the total gene signal, while each mRNA value is the log(REDsignal/GREENsignal) per feature (processed signals used, base 10). Of the 36 samples, 30 (24 HCV+ and 6 HCV-) samples have paired miRNA and mRNA expression profiles. Experiments were conducted on all samples using four technical replicates with the exception of sample28, sample33 and sample35, for which only three replicates were used. It is very costly for wet-lab experiments to obtain such a paired miRNA and mRNA expression data set. To our best of our knowledge, the paired data set used in this work is the largest microarray paired data set in the existing literature.

### Discovery of strong discriminatory rules

Given a data set containing two classes of samples (positive and negative), we discover strong rules in the form: ${⋂}_{i=1‘}^{k}a_{i}\leq x_{i}\leq b_{i}$, where *x_i _*represents a miRNA or a mRNA, [*a_i_, b_i_*] is the expression range of *x_i_*. If every positive sample's expression profile satisfies (falls into) the *k *specific expression ranges, but none of the negative sample profiles satisfies, then we say it is a 100%-frequency rule to differentiate the positive samples from the negative samples. The complete form of this rule is denoted by ${⋂}_{i=1}^{k}a_{i}\leq x_{i}\leq b_{i}\to positive\left(100\%\right)$. This suggests that if the expression of every *x_i _*is between *a_i _*and *b_i _*for a HCV test sample, then this test sample is very likely to be a positive sample. Similarly in this work, we also define the 100%-frequency rule to differentiate negative samples from positive samples. This study identifies simple 2-miRNA 100%-frequency rules (i.e., *k *= 2) to capture differentially expressed miRNAs and the miRNA expression changes in HCV infection. We do not identify 3-miRNA 100%-frequency rules or the rules involving more than 3 miRNAs (i.e., *k *> 3). The stringent 100%-frequency may be unnecessary for other data sets as such distinction may not exist. Therefore, this frequency requirement can be relaxed for other studies.

### Rule-based identification of miRNA-mRNA regulatory modules

**Figure 2:**
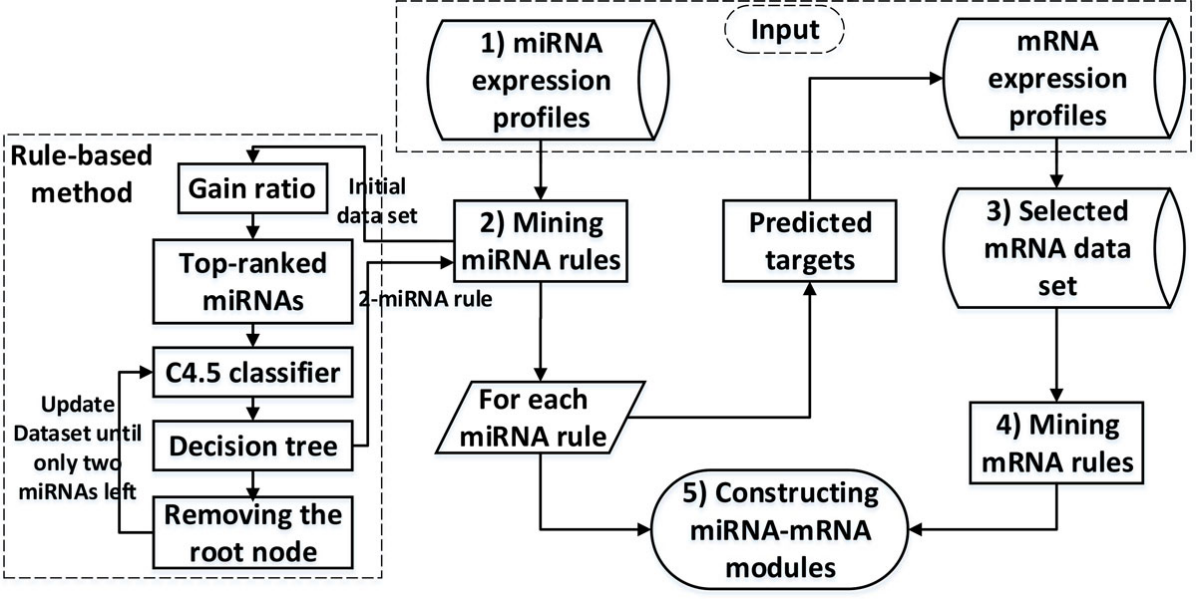
**Computational steps for the identification of miRNA-mRNA regulatory modules**. 1) Collection of miRNA expression profile data set. 2) Discovery of discriminatory rules from the miRNA expression data set using our rule discovery algorithm. 3) Construction of a selected and relevant mRNA expression data set. 4) Discovery of discriminatory rules from the relevant mRNA data set. 5) Identification of candidate miRNA-mRNA regulatory modules by combining the miRNAs and mRNAs in the discovered rules.

We take the following steps to detect miRNA-mRNA regulatory modules. The first step is to use rule discovery to identify differentially expressed miRNA rules of 100%-frequency. Then for every miRNA in each rule, we obtain its predicted mRNA targets by searching for a public database [18]. We then construct a selected mRNA data set for each rule consisting of all the samples but only those predicted target mRNAs presented in the original mRNA data set. We subsequently detect mRNA rules of 100% frequency from this selected mRNA data set. The mRNA rules and their miRNA rule are then combined to form a miRNA-mRNA regulatory module. Our method is summarized in Figure 2 and detailed in the following subsections.

#### Rule discovery from miRNA expression data

We rank all of the 470 miRNAs using the gain ratio criteria [21,22] through the Weka 3.6 software package [23]. The top-ranked miRNAs (the most significant miRNAs) are then extracted to construct a new data set. We take a committee tree approach to detect 100%-frequency rules from this new data set, and to generate a committee of decision trees. We use the implementation of the C4.5 algorithm [21] in the R software package(RWEKA) to construct the tree committee. The first tree is derived based on the above miRNA data set. To derive the second tree, we change the data set by removing the root node of the first decision tree. This process is repeated until the data set has only two miRNAs left. If all of the training samples can be correctly classified by a rule in one of these trees, then this rule is a 100%-frequency rule. As mentioned, this work focuses on only 2-miRNA 100%-frequency rules as differentially expressed miRNAs for the simple diagnosis of HCV infection.

All the 100%-frequency rules are evaluated by Euclidean distance [24] and the average area under receiver operating characteristic (ROC) curves (AUCs) in the 10-fold decision tree cross-validation to determine their significance. Euclidean distance of a 100%-frequency rule indicates the separation extent between the HCV+ and HCV- samples. The separation extent is measured by the shortest pair-wise Euclidean distance of the HCV+ and HCV- samples (i.e., the Max-Min distance). The wider the separation is, the more reliable is the rule. 100%-frequency rules with a wide separation distance are of our interest for a further investigation.

#### Rule discovery from the mRNA data set

The systematic function of a miRNA is ultimately defined by its interaction with its target mRNAs or genes. We thus investigate the co-expressed miRNAs in each rule and their corresponding target mRNAs that are corporately involved in HCV infections. For each rule, we obtain computationally predicted target mRNAs through the Targetscan database [18]. Using these predicted target mRNAs and their corresponding expression profiles from the original mRNA data, we apply data mining techniques below to discover the rules of mRNA targets.

Given a dataset *D *with the class label set *C *(e.g., positive and negative), so we detect rules for each *c *∈ *C*. There may be more than one rule for each *c *∈ *C*, we use several rounds of rule analysis to detect the rules. In each round, we detect a rule for each *c *∈ *C*. We enumerate every attribute *x_i _*to get its expression range *a_i _*and *b_i_*, and calculate the compactness *p *= *N_c_*/*N *where *N *or *N_c _*is the number of all samples or *c*'s samples in the expression range. Then, *a_i _*≤ *x_i _*≤ *b_i _*with the highest compactness is added to the rule. This process is repeated until (i) *p *= 100% (a rule for *c *is detected), or (ii) *p *cannot be improved but is still below 100% (there is no rule for *c*). In the selected mRNA expression dataset used in this work, in each round of rule analysis, we detect two rules: one for HCV+ and the other for HCV-. For next round, all mRNAs in the discovered rules beforehand are not considered, and the rule analysis is performed again to detect more rules. The whole process is terminated until none of the classes has a rule. This computationally heavy method is used at this step, because it is hard to identify the significant mRNAs with high gain ratio among tens of thousands of mRNAs by the tree-based analysis, but this computational heavy method can work out many 100%-frequency rules from the mRNA data sets.

#### Rule-based miRNA-mRNA regulatory modules

We group all of its mRNA rules of 100%-frequency for each miRNA rule. A miRNA-mRNA regulatory module is formed by using a bipartite graph representation [25], in which all the mRNAs in these rules comprise the mRNA partite, while the miRNAs are placed at the miRNA partite. To show the significant part of the modules and to assess the modules in the validation, we focus on the top four mRNA rules: two rules for classifying HCV+ samples and two rules for HCV-. We refer to these miRNAs and those mRNAs in the top four rules as significant components of the miRNA-mRNA regulatory module.

We also review the existing empirical literature to assess the biological importance of the regulatory modules. Furthermore, Pearson's correlation coefficient is calculated to detect the relationships (positive or negative correlation) between the miRNAs and mRNAs.

## Results

### Discriminatory rules from the miRNA expression data

On the original miRNA data set of the 36 samples and 470 miRNAs, the gain ratio method selects 21 top-ranked miRNAs as the most significant miRNAs for the distinction between the HCV+ and HCV- samples. Each of these 21 miRNAs has a gain ratio >0.5. The other miRNAs have a gain ratio ≤ 0.5 and thus were not considered for further study here. Statistical analysis is also carried out using the two-sided student's t-test and the statistical significance is set as P < 0.05 (Table 1).

**Table 1:** The top-ranked miRNAs with gain ratio larger than 0

miRNA	Rank	p-value	miRNA	Rank	p-value
miR-202	1	2.072e-08	miR-519e*	12	4.222e-04
miR-601	2	1.060e-05	miR-526b	13	8.246e-04
miR-498	3	6.196e-09	miR-345	14	9.802e-05
miR-557	4	1.148e-05	miR-17-3p	15	2.927e-05
miR-34a	5	1.767e-02	miR-520a	16	0.276
miR-493-3p	6	3.127e-06	miR-452	17	4.170e-05
miR-214	7	4.629e-03	miR-501	18	4.328e-07
miR-184	8	1.470e-06	miR-130a	19	7.261e-04
miR-129	9	3.752e-03	miR-34b	20	1.278e-02
miR-765	10	1.243e-08	miR-221	21	4.622e-02
miR-210	11	1.668e-08			

On the data set of the above 21 miRNAs and all of the 36 samples, a total of nine 100%-frequency rules covering 10 miRNAs are derived through our committee tree approach. Each of these rules can classify the 36 samples into HCV+ or HCV- without any misclassification. An example of these miRNA rules is related to miR-557 and miR-214. The rule is that: every HCV+ sample's expression profile satisfies the two miRNAs' specific expression ranges: 8.94 ≤ *miR *− 557 ≤ 43.53 ∩ 95.54 ≤ *miR *− 214 ≤ 1057.51, but none of the HCV- samples satisfies these two expression ranges. The minimum Euclidean distance separating the two classes of samples for each rule and the average AUCs in the 10-fold decision tree cross-validation are also calculated. As shown in Table 2 the rule consisting of miR-557 and miR-214 has the maximum distance and maximum AUC.

**Table 2:** The target mRNAs and their rules for each miRNA rule.

Rule ID	Euclidean distance	Average AUC	#mRNA in dataset	Class^1^	#rules^2 ^in HCV+	#rules^3 ^in HCV-	#mRNAs in all rules	#mRNAs^4 ^in top rules
R1	4.8946	0.9323	300	HCV+	2	14	110	15
R2	3.2888	0.9323	517	HCV+	2	21	159	12
R3	2.5160	0.9323	329	HCV+	2	14	85	12
R4	2.3360	0.8889	247	HCV+	2	11	75	12
R5	0.2256	0.8681	184	HCV+	2	5	41	13
R6	1.6425	0.9115	650	HCV-	8	34	269	10
R7	1.2757	0.8750	398	HCV-	7	28	227	10
R8	2.6420	0.9028	186	HCV-	2	6	55	20
R9	0.9806	0.8958	289	HCV-	1	12	97	11

R1: miR-557 and miR-214; R2: miR-34a and miR-214; R3: miR-493-3p and miR-214; R4: miR-214 and miR184; R5: miR-184 and miR-210; R6: miR-129 and miR-765; R7: miR-765 and miR-210; R8: miR-210 and miR-452; R9: miR-452 and miR-17-3p. ^1 ^: the miRNA rule defines a region covering all samples of a class.

^2^(^3^): the number of mRNA rules, each of which defines a region covering all samples of HCV+(HCV-).

^4^: the number of mRNAs in the top four mRNA rules: the top two mRNA rules in HCV+ (one rule is used if it is the only mRNA rule in HCV+) and another top two rules in HCV-.

### Rules from the mRNA expression data

For each miRNA rule, the predicted mRNA targets from TargetScan are used to narrow down the original mRNA data set to a relevant mRNA data set for mRNA rule discovery. As shown in Table 3 some of the predicted targets (mRNAs) of a miRNA are not in the list of the probes used in the original mRNA expression data set (Table 2). Therefore, the mRNA expression profiles of only those targets (mRNAs) of the miRNAs in the probe list are used for the rule discovery (forth column of Table 2). We note that the miRNAs involved in each rule may have common targets. For example, miR-557 and miR-214 have two common targets. On the 9 new mRNA data sets each for one miRNA rule, many 100%-frequency rules were mined by our proposed rule mining method (Table 2). In detail, we identified 28 mRNA rules for HCV+ and 145 mRNA rules for HCV- covering a total of 1118 mRNAs for all of the 9 miRNA rules (Table 2). Lastly, the top 4 rules, 2 from HCV+ (one rule is used if it is only one mRNA rule in HCV+) and 2 from HCV- are chosen as differentially expressed mRNAs for the subsequent miRNA-mRNA regulatory module study.

**Table 3:** Predicted targets in the TargetScan and those targets common in our data set.

miRNA	mRNA targets	
	
	Predicted by TargetScan	in our used data set
miR-557	97	78
miR-214	301	224
miR-34a	387	293
miR-493-3p	131	105
miR-184	28	23
miR-129	320	236
miR-765	1105	414
miR-452	32	25
miR-210	218	161
miR-17-3p	353	264

### A miRNA-mRNA regulatory interaction network

**Figure 3:**
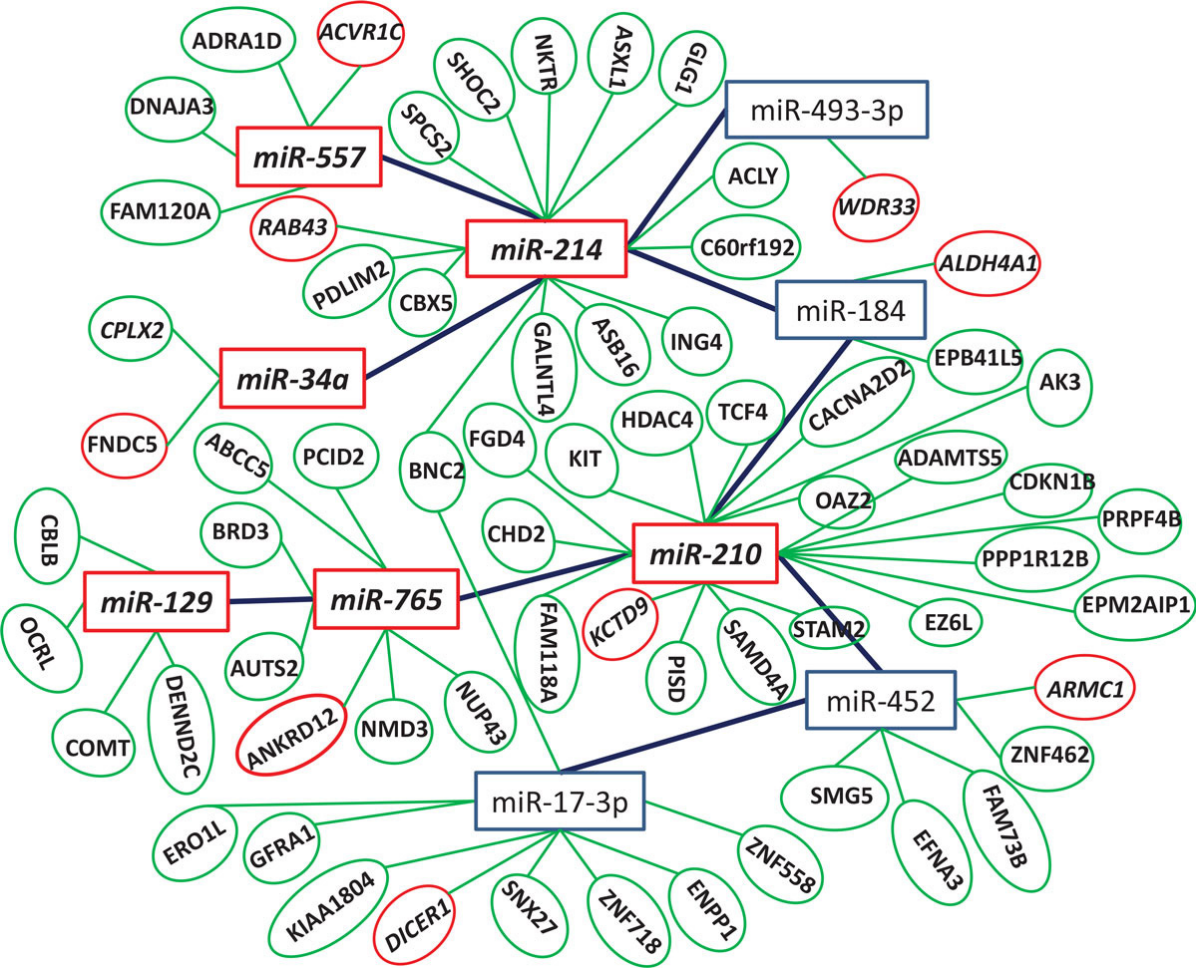
**A miRNA-mRNA regulatory interaction network**. There is an edge between two miRNAs if they are components of a miRNA rule. The edge between a miRNA and a mRNA represents a regulation of the miRNA for its target. Six miRNAs (miR-214, miR-34a, miR-129, miR-765 and miR-210) and 9 mRNAs (ACVR1C, RAB43, FNDC5, WDR33, ALDH4A1, ANKRD12, KCTD9, ARMC1 and DICER1) all in red are confirmed by literature work.

The above detected miRNA rules and significant mRNA rules are merged to form 9 miRNA-mRNA regulatory modules. These 9 miRNA-mRNA regulatory modules are then integrated to form a bigger miRNA-mRNA regulatory network (Figure 3). Many miRNAs and mRNAs with bold in these modules are related to diseases, in particular hepatocellular carcinoma, as supported in the literature (Table 4).

**Table 4:** All target mRNAs of miRNAs in HCV+ and HCV- modules.

miRNAs	Targeted mRNAs	modules
**miR-557**	ADRA1D, **ACVR1C**, DNAJA3, FAM120A	HCV+
**miR-214**	ASB16, GALNTL4, CBX5, BNC2, PDLIM2,**RAB43**, SPCS2, NKTR, ASXL1, ACLY,C6orf192, ING4, GLG1, SHOC2	HCV+
**miR-34a**	CPLX2, **FNDC5**	HCV+
miR-493-3p	**WDR33**	HCV+
miR-184	EPB41L5, **ALDH4A1**	HCV+
**miR-129**	CBLB, OCRL, COMT, DENND2C	HCV-
**miR-765**	ABCC5, BRD3, **ANKRD12**, AUTS2, PCID2, NMD3, NUP43	HCV-/+
**miR-210**	FGD4, HDAC4, CACNA2D2, OAZ2, ADAMTS5, AK3, CDKN1B, EPM2AIP1, PPP1R12B, PRPF4B, STAM2, EZ6L, SAMD4A, PISD, **KCTD9**, FAM118A, CHD2, KIT, TCF4	HCV-
miR-452	**ARMC1**, ZNF462, EFNA3, SMG5, FAM73B	HCV-
miR-17-3p	BNC2, **DICER1**, GFRA1, KIAA1804, ENPP1, ZNF558, ERO1L, SNX27, ZNF718	HCV-

**Figure 4:**
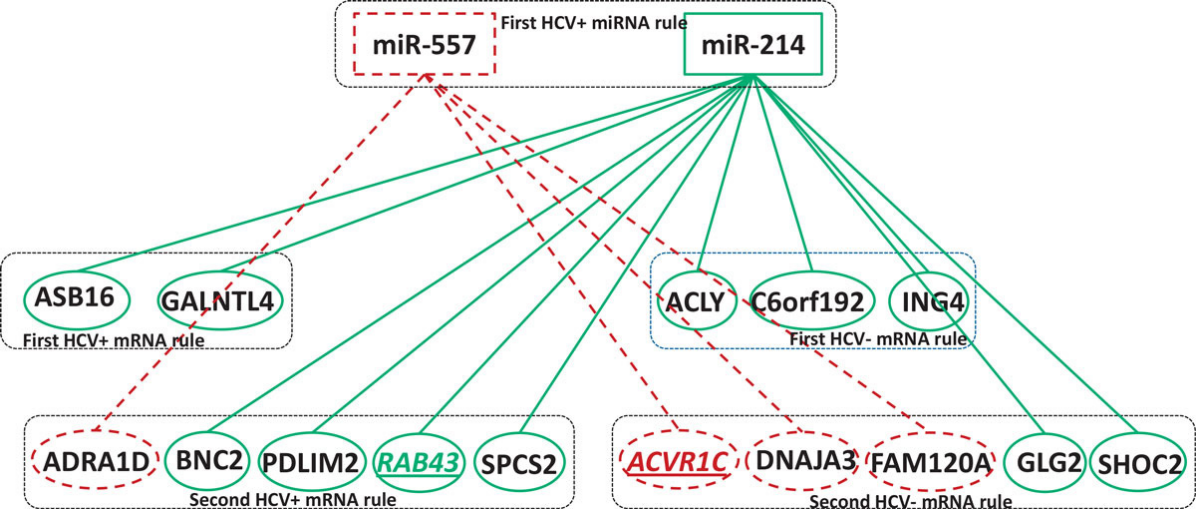
**The regulatory module inferred from the first miRNA rule and its corresponding mRNAs**. miR-557 and miR-214, the miRNAs of the first HCV+ miRNA rule are placed at the up panel. Four mRNA rules are identified and their mRNAs are placed at in the middle and bottom panels. The edges linking miR-214 and its mRNA targets are in solid lines, while the edges linking miR-557 and its mRNA targets are in dashed lines. The confirmed target mRNAs are also highlighted with an underline.

**Figure 5:**
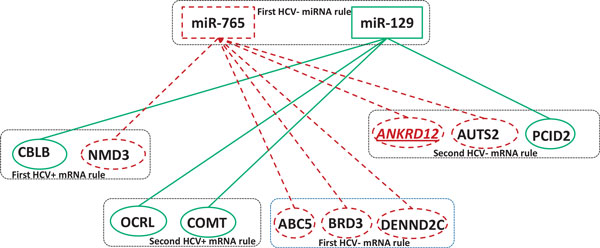
**The regulatory module inferred from the first HCV- rule consisting of miR-129 and miR-765**. In this module, miR-765 targets 6 mRNAs and miR-129 regulates 4 mRNAs. ANKRD12, a target of miR-765, is validated to be associated with chronic liver disease by existing works.

The numbers of mRNAs in these significant modules are shown in the last column of Table 2. Figure 4 and 5 show two examples of these significant modules, and all the miRNAs in bold and the mRNAs with underline and italics can be confirmed by the literature. The validation results are presented below:

• In the module for miR-557 and miR-214 (Figure 4), miR-557 has been reported as a novel candidate biomarker for hepatocellular carcinoma [26], and miR-214-5p has been shown to up-regulate in human and mouse livers in a fibrosis progression-dependent manner. The expression of miR-214-5p increased during the culture-dependent activation of mouse primary stellate cells and was significantly higher in stellate cells than in hepatocytes [27]. As miR-214 and miR-557 expression patterns in hepatocellular carcinoma are tissue specific, they can both serve as novel biomarkers for chronic liver diseases. Meanwhile, a target mRNA *ACVR1C *of miR-557 is also associated with a reduction in HCV-infected cells [28], while the target mRNA *RAB43 *of miR-214, a key RAB to maintain a functional Golgi complex in human cells [29], has been found to interact with HCV NS5A proteins [30] and can also mediate the replication of HCV [29].

• In the module of miR-34a and miR-214, besides the confirmed miR-214 and its mRNA *RAB43*, miR-34a has been reported to upregulate in both liver fibrosis and hepatocellular carcinoma, and serum levels of miR-34a are significantly higher in chronic hepatitis C infection patients than in controls [31]. In addition, its target mRNA Fibronectin (*FNDC5 *) was down-regulated and associated with hepatic fibrosis [32].

• In the module of miR-493-3p and miR-214, the sole target mRNA *WDR33 *of miR-493-3p has been found to result in increased viral infection with two or more siRNAs [33].

• In the module of miR-184 and miR-214, a mRNA *ALDH4A1 *of miR-184 was believed to contribute to HBV- or HCV- induced liver [34].

• In the module of miR-129 and miR-765 (Figure 5), miR-129 has been strongly believed to be involved in the significant dysregulation in hepatocellular carcinogenesis [26,35], and miR-765 is one of promising candidate miRNA biomarkers to detect hepatocellular carcinoma among hepatitis C virus patients [36]. Meanwhile, mRNA *ANKRD12 *of miR-765 is involved in one of the important roles of host miRNAs in regulating the liver-specific HCV [37].

• In the module of miR-765 and miR-210, besides the validation of miR-765 and its target mRNAs above, miR-210 was upregulated in HBV-producing HepG2.2.15 cells compared to parental HepG2 cells, and identified to suppress hepatitis B virus [38]. In addition, a target mRNA (*KCTD9*) of miR-210 has been found to make contribution to liver injury [39].

Other mRNAs in these modules are also confirmed to be involved in hepatocellular diseases. For example, a mRNA of miR-452, *ARMC1 *is up-regulated and frequently amplified in human hepatocellular carcinoma [40]. Target mRNA *DICER1 *of miR-17-3p, a component of the RNAi machinery, can markedly reduce HCV production and intracellular HCV RNA levels [41].

All these validation results suggest that the identified modules are closely related to hepatocellular carcinoma and are novel knowledge for understanding the miRNA-mRNA regulation in the host responses and pathogenesis of HCV infection.

### Many-to-many miRNA-mRNA regulatory modules

**Figure 6:**
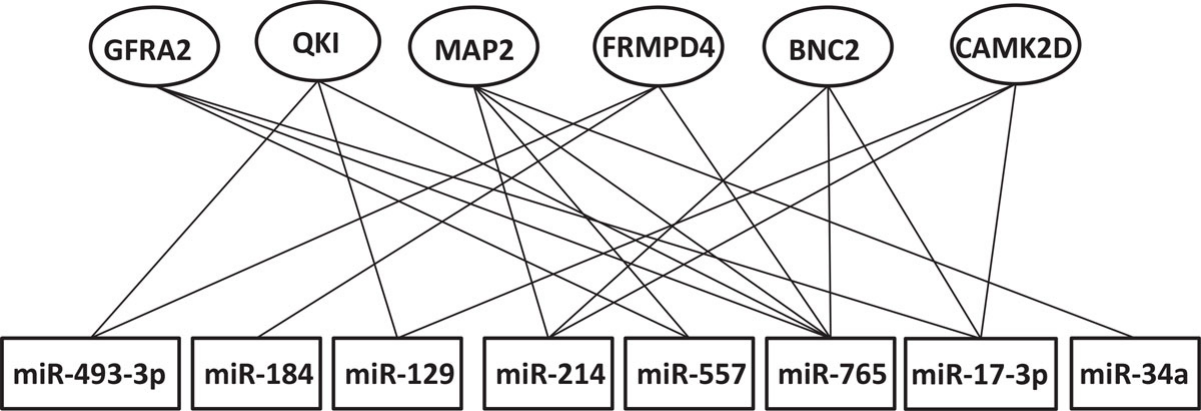
**The many-to-many relationship between some mRNAs and miRNAs identified in our modules**. One mRNA are targeted by many miRNAs and one miRNA can regulate many mRNAs

The big regulatory module (Figure 3) is a miRNA-mRNA interaction network integrated from the 9 simple regulatory modules corresponding to the 9 miRNA rules. A many-to-many miRNA-mRNA regulatory module usually consists of a cohort of miRNAs and a set of their target mRNAs, in which a target mRNA is regulated by multiple miRNAs, and a miRNA has multiple mRNAs as its target. We specially examined those many-to-many miRNA-mRNA regulatory modules in which mRNAs are targeted by at least 3 miRNAs. Figure 6 shows such an example.

This regulatory module contains 8 miRNAs and 6 target mRNAs. The literature shows that the miRNAs in this regulatory module are causally connected to human hepatocellular carcinoma or related diseases. For example, miR-129, miR-214 and miR-34a are found to associate with human hepatocellular carcinoma [26,31,35,42]. Mature miR-184 of over-expression can act as an oncogene in the antiapoptotic and proliferative processes of tongue Squamous Cell Carcinoma [43]. miR-129 can regulate multiple tumor cell lines and primary tumors including medulloblastoma, undifferentiated gastric cancers, lung adenocarcinoma, endometrial cancer and colorectal carcinoma through down-regulating *CDK6 *expression [44]. miR-34a can act as a tumor suppressor gene in a broad range of tumors including breast cancer, lung cancer, colon cancer, kidney cancer, bladder cancer and pancreatic carcinoma cell lines [45]. The mRNAs targeted by the miRNAs in this regulatory module are also engaged with cancer. Tumor suppressor *QKI *(the common target of miR-493-3p, miR-129 and miR-765) is expressed at significantly low levels in most of the gastric cancer tissues [46]. *MAP2 *has been reported to be involved with malignant oral cancer tissues by playing important roles in neuronal and non-neuronal development [47].

### Negatively and positively regulated mRNAs by multiple miRNAs

The miRNA-mRNA expression relationships in the above many-to-many regulatory module were further assessed by analysing the Pearson's correlation coefficients of the 19 paired miRNA and mRNA expression levels of the 30 patients (i.e., the 19 edges in Figure 6). These coefficients are shown in Table 5. As expected, most of these relationships are negative. For example, *QKI*, *MAP2 *and *BNC2 *have an inverse expression relationship with all of their regulator miRNAs. *FRMPD4 *is also negatively correlated with their regulators except for miR-493-3p. *CAMK2D *has a random correlation with miR-129 and miR-214, but it is negatively correlated with miR-17-3p.

**Table 5:** Pearson's correlation coefficients between the miRNAs and mRNAs in the many-to-many regulatory module.

	GFRA2	QKI	MAP2	FRMPD4	BNC2	CAMK2D
miR-493-3p	-	-0.68	-	0.12	-	-
miR-184	-	-	-	-0.05	-	-
miR-129	-	-0.71	-	-	-	0.01
miR-214	-	-	-0.15	-	-0.01	0.03
miR-557	0.18	-	-0.01	-	-	-
miR-765	0.21	-0.44	-0.02	-0.04	-0.10	-
miR-17-3p	0.26	-	-	-	-0.13	-0.13
miR-34a	-	-	-0.05	-	-	-

(Figure 8).'-' indicates the mRNA (in a column) is not the target of the miRNA (in a row)

**Figure 7:**
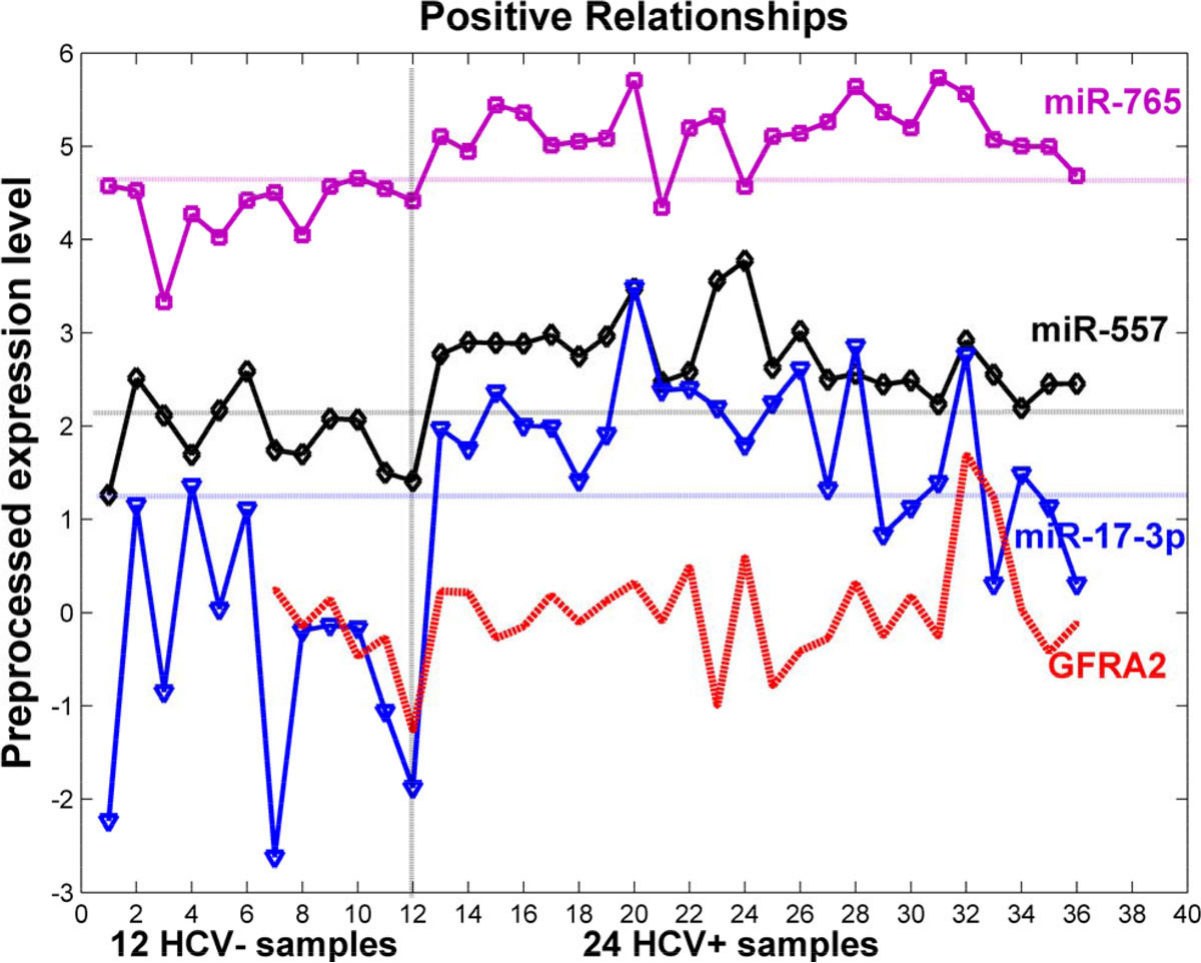
**The positive expression relationship between GFRA2 mRNA and miR-557, miR-765, and miR-17-3p**. The expression levels of the three miRNAs are preprocessed in the log scale, and the expression levels of *GFRA2 *are expanded by 10 times. The three miRNAs all have a high gain ratio, separating the HCV+ and HCV- samples very well.

One of our novel findings is a positive regulatory relationship between a mRNA and multiple miRNAs. As can be seen from Table 5, *GFRA2 *has a clear positive relationship with the expression of all of miR-557, miR-765 and miR-17-3p with Pearson's correlation coefficients 0.18, 0.21, and 0.26 respectively. Figure 7 details these positively regulated expression levels of *GFRA2 *of the 30 patients in comparison with the expression levels of the three miRNAs. As indicated by the gain ratios shown in Table 1, the expression levels of each of these three miRNAs are able to separate these HCV+ and HCV- samples (also seen from the horizontal lines in Figure 7). It is the expression change of these three miRNAs that leads to a positive expression change of *GFRA2 *between the two classes of patients.

**Figure 8:**
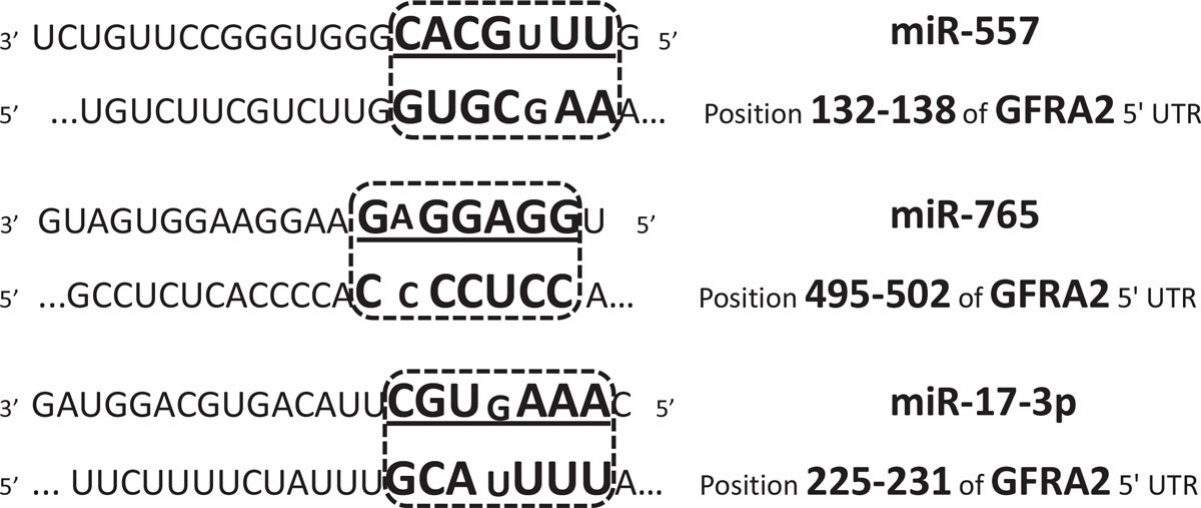
**The partial complementary sequence pairing**. The 5' UTRs of *GFRA2 *contains the seed sites of miR-557, miR-765 and miR-17-3p. The mismatched base pairs are shown in smaller font.

The sequence matching between these miRNAs and *GFRA2 *was also studied. Ørom *et al*. reported that the binding of miR-10a at the 5' UTRs of ribosomal protein (RP) mRNAs can promote their translational enhancement instead of repression [12]. We attempted to verify whether the 5' UTR of *GFRA2 *has a full or partial complementary sequence pairing with the seed region of miR-557, miR-765 or miR-17-3p. The fact is that the seed region of these three miRNAs is complementary to the 5' UTR of *GFRA2 *with just one mismatched pair. In detail, the seed region of miR-557 matches the positions from 132 to 138 of *GFRA2 *5' UTRs, the seed region of miR-17-3p matches from 225 to 231, and the seed region of miR-765 matches from 495 to 502 (Figure 8). Therefore, it is likely that these three miRNAs bind at the 5' UTR end of *GFRA2 *mRNA to enhance its translation for a positive regulation.

The statistical significance of this sequence complementarity in the defined manner (which includes a mismatch) was analyzed using a Markov Model (MM) [48]. Based on first-order Markov model [49], the complementary significance was assessed by computing a probability (P) for each miRNA-5' UTR pair. It is an approximate probability that a complementary to the miRNA seed is found in the corresponding 5' UTR. The lower the P is, the higher the chances that the 5' UTR is a functional target. The length of the 5' UTR of *GRFA2 *mRNA is 675, composed of 151 purine bases adenine (A), 182 guanine (G), 161 the pyrimidine bases uracil (U), and 181 cytosine (C). The number of nucleotide in the miRNA seed region is 7. The transition matrix is shown as in Table 6. The complementary probability of the sequence matching between the seed region of the three miRNAs (miR-557, miR-765 and miR-17-3p) and the 5' UTRs of *GFRA2 *are 1.337e-05, 1.488e-04, and 1.133e-04 respectively which all imply a strong indication of a functional target.

**Table 6:** Transition probability of two adjacent bases in the 5' UTRs of GFRA2.

	A	G	U	C	Sum
A	33 (0.219)	50 (0.331)	32 (0.212)	36 (0.238)	151 (1.000)
G	60 (0.330)	60 (0.330)	20 (0.110)	42 (0.230)	182 (1.000)
U	22 (0.137)	33 (0.205)	62 (0.385)	44 (0.273)	161 (1.000)
C	36 (0.199)	38 (0.210)	47 (0.260)	60 (0.331)	181 (1.000)

Sum	151 (0.885)	181 (1.076)	161 (0.967)	182 (1.072)	675 (4.000)

To the best of our knowledge, the expression relationship between *GFRA2 *and any of the three miRNAs has not been studied before in spite of intensive separate research for this mRNA or these miRNAs. *GFRA2 *is a member of the *GDNF *receptor family encoding *GDNF *family receptor alpha-2 protein. *GFRA2 *is also a glycosylphosphatidylinositol (GPI)-linked cell surface receptor for both the Glial cell line-derived neurotrophic factor (GDNF) and neurturin (NTN) [50], and it can affect the activation of the RET tyrosine kinase receptor [51]. *GFRA2 *is a candidate gene for RET-associated diseases. Brain-derived neurotrophic factor in patients has been found to be related with Chronic Hepatitis C [52]. Independent of the research on *GFRA2*, miR-557 [26], miR-765 [36] and miR-17-3p [53] all have been reported to associate with hepatocellular carcinoma. This suggests that the binding and interaction of mRNA *GFRA2 *with miR-557, miR-765, or miR-17-3p, or with their combinations is a new research area, worthy of comprehensive investigation by wet-lab experiments.

**Figure 9:**
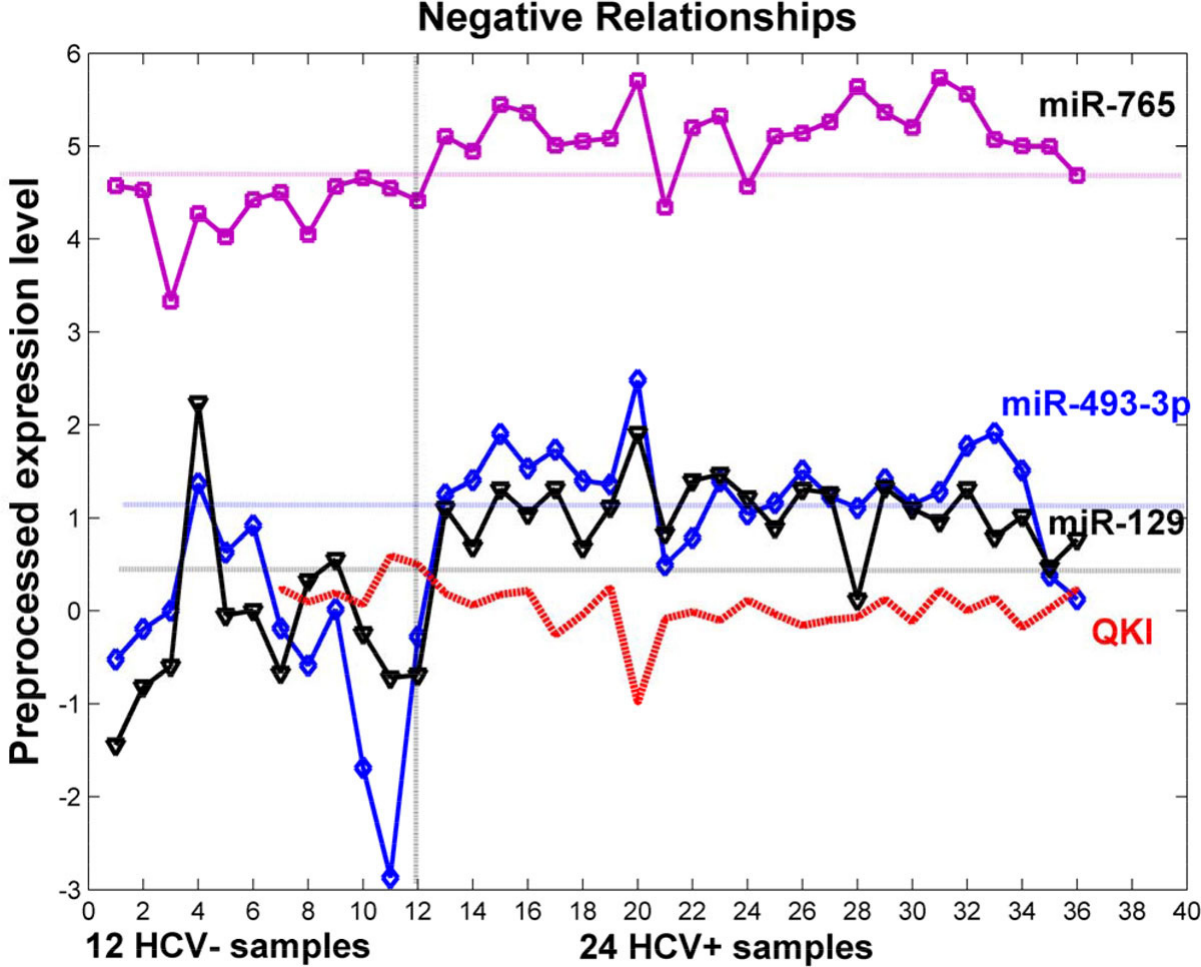
**An example of the negative expression relationship**. An negative relationship between the *QKI *mRNA and miR-493-3p, miR-129, and miR-765. The expression levels of the three miRNAs are preprocessed in the log scale. The three miRNAs all have a good gain ratio, separating the HCV+ and HCV- samples very well.

We also closely examined a strong negative regulatory relationship, shown in Figure 6. This regulatory relationship is between *QKI *mRNA and multiple miRNAs miR-493-3p, miR-129 and miR-765 (see Figure 9). The seed matching sequence of miR-129 is located within the 3' UTRs end of *QKI*. But, the 5' UTRs of *QKI *mRNA does not contain the miR-129 complementary seed site. It is believed that miR-129 binds at the 3' UTRs end of *QKI *mRNA to down regulate its translation.

Pearson's correlation coefficients were similarly examined for the literature-confirmed miRNAs and their corresponding mRNAs in Figure 3. It was found that miR-17-3p and *DICER1 *mRNA have a strong negative regulatory relationship (Pearson's correlation coefficient: -0.53). A protein possessing an RNA helicase motif can be encoded by *DICER1 *gene. The encoded protein functions as a ribonuclease and is required to produce the active small RNA component that represses gene expression, which may affect the biogenesis of miRNA [54].

**Figure 10:**
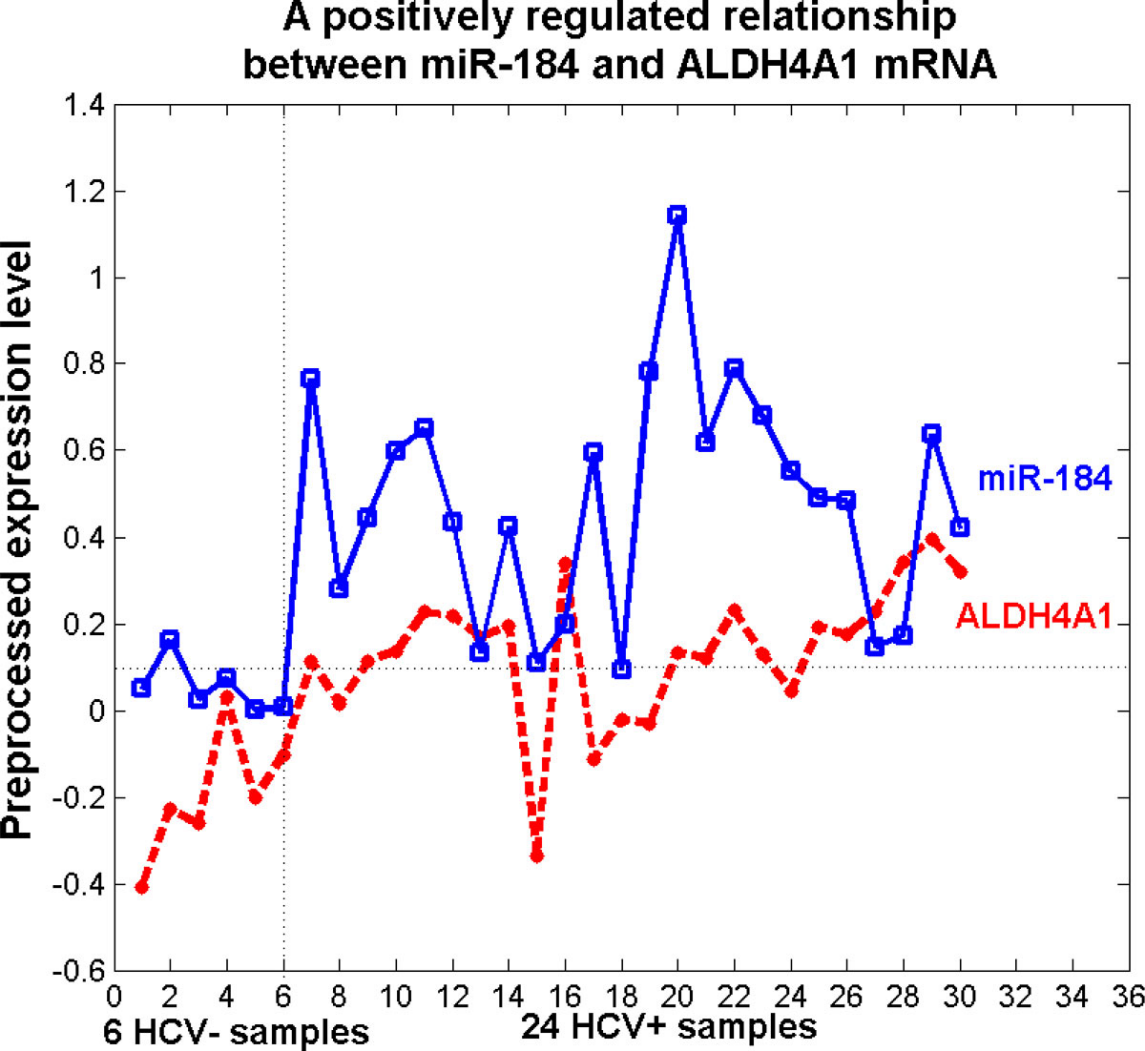
**An example of the positive expression relationship**. An positive realtionship between the *ALDH4A1 *mRNA and miR-184. The expression levels of miR-184 is preprocessed by dividing 10 and has a good gain ratio, classifying the HCV+ and HCV- samples very well.

As found by this work, the strongest positively regulated relationship is between miR-184 and *ALDH4A1 *mRNA. Its Pearson's correlation coefficient is 0.43 (Figure 10). The *ALDH4A1 *mRNA is up-regulated in late HCV cirrhosis [55] and HBV pathogenesis [34]. In Drosophila, a luciferase reporter assay has shown that miR-184 can target some mRNAs in the protein coding region [56]. We found that the seed region of miR-184 is complementary to the coding region or to the 5' UTR of *ALDH4A1 *with just one mismatched pair. The seed region of miR-184 matches the positions from 705 to 711 of *ALDH4A1 *'s coding region or with the positions from 257 to 263 at *ALDH4A1 *5' UTR. Based on this evidence, the miRNA-184 target sites in 5' UTRs or coding region may make significant contribution to miR-184 mediated regulation. The functionality of miR-184 when binding at the 5' UTR or the coding region of *ALDH4A1 *deserves thorough investigation to expand the current research on the 3' UTR only.

We also found other evidence supports that ectopic expression of miRNA-184 leads to neuroblastoma cell growth arrest and apoptosis, miRNA-184 expression is repressed in human neuroblastoma tissues, and low levels of miRNA-184 expression in human neuroblastoma tissues correlate with poor patient survival [57]. We next made use of the publically available Versteeg microarray gene expression dataset http://r2.amc.nl, which contained both gene expression levels and patient prognosis. Kaplan-Meier analysis showed that low levels of ALDH4A1 mRNA expression in human neuroblastoma tissues positively correlated with poor patient prognoses (p ¡ 0.01). As low levels of miR-184 in human neuroblastoma tissues also correlates with poor patient survival, the data suggest that miR-184 is likely to positively regulate ALDH4A1 expression in tumours.

In addition, we also checked our discovery results in the miRTarbase database [58,59] and starBase database [60,61], an interaction between miR-214 and *ING4 *mRNA (Table 4) in the miRTarbase database is experimentally validated by reporter assay and quantitative polymerase chain reaction (qPCR). Another interaction is confirmed between hsa-miR-129-5p (previous ID: hsa-miR-129) and *QKI *in the starBase database, with the highest Pearson's Correlation Coefficient in Table 5.

## Conclusion

In this work, we have proposed rule-based methods for the discovery of miRNA-mRNA regulatory modules in HCV infection. We followed the biological principle that inverse expression relationships and positively regulated miRNA-mRNA pairs can both exist in many-to-many regulatory modules. We detected 100%-frequency rules from the most differentially expressed miRNAs and then mined 100%-frequency rules from the relevant target mRNAs expression data for each miRNA rule. We integrated the miRNA rules and their mRNA rules to construct miRNA-mRNA regulatory modules. Many detected miRNAs and mRNAs can be supported by recent work in the literature. We also detected novel positive and inverse regulatory relationships. For example, mRNA *GFRA2 *is positively regulated by multiple miRNAs miR-557, miR-765 and miR-17-3p which all likely bind at the 5' UTR end of *GFRA2*. The detected miRNA-mRNA regulatory modules will provide new insights into the regulation of host responses and the pathogenesis of HCV infection. We conclude that our rule discovery method is useful for integrating binding information and expression profile for identifying HCV miRNA-mRNA regulatory modules and can be applied to the study of the expression profiles of other complex human diseases.

## Abbreviations

HCV: Hepatitis C virus; NCBI: National Center for Biotechnology Information; GEO: Gene expression omnibus; ROC: Receiver operating characteristic; AUCs: Area under ROC curves; RP: Ribosomal protein; MM: Markov Model; P: probability; A: adenine; G: guanine; U: uracil; C: cytosine; GPI: glycosylphosphatidylinositol; GDNF: Glial cell line-derived neurotrophic factor; NTN: neurturin.

## Competing interests

The authors declare that they have no competing interests.

## Authors' contributions

RS carried out the experiments and drafted the initial manuscript. QL contributed to the design of the algorithms. JL initiated and supervised the study, and revised the manuscript. All authors read and approved the final manuscript.
